# Parental diabetes status reveals association of mitochondrial DNA haplogroup J1 with type 2 diabetes

**DOI:** 10.1186/1471-2350-10-60

**Published:** 2009-06-18

**Authors:** Jeanette Feder, Ofer Ovadia, Ilana Blech, Josef Cohen, Julio Wainstein, Ilana Harman-Boehm, Benjamin Glaser, Dan Mishmar

**Affiliations:** 1 Department of Life Sciences, Ben-Gurion University of the Negev, Beer-Sheva, Israel; 2 The National Institute of Biotechnology (NIBN), Ben-Gurion University of the Negev, Beer-Sheva, Israel; 3 Endocrinology and Metabolism Service, Hadassah-Hebrew University Medical School, Jerusalem, Israel; 4 Maccabi Health Services, Diabetes Clinic, Rishon LeZion, Israel; 5 Wolfson Medical Center, Holon, Israel; 6 Soroka Hospital, Ben-Gurion University of the Negev, Beer-Sheva, Israel; 7 Israel Diabetes Research Group (IDRG), Israel

## Abstract

**Background:**

Although mitochondrial dysfunction is consistently manifested in patients with Type 2 Diabetes mellitus (T2DM), the association of mitochondrial DNA (mtDNA) sequence variants with T2DM varies among populations. These differences might stem from differing environmental influences among populations. However, other potentially important considerations emanate from the very nature of mitochondrial genetics, namely the notable high degree of partitioning in the distribution of human mtDNA variants among populations, as well as the interaction of mtDNA and nuclear DNA-encoded factors working in concert to govern mitochondrial function. We hypothesized that association of mtDNA genetic variants with T2DM could be revealed while controlling for the effect of additional inherited factors, reflected in family history information.

**Methods:**

To test this hypothesis we set out to investigate whether mtDNA genetic variants will be differentially associated with T2DM depending on the diabetes status of the parents. To this end, association of mtDNA genetic backgrounds (haplogroups) with T2DM was assessed in 1055 Jewish patients with and without T2DM parents ('DP' and 'HP', respectively).

**Results:**

Haplogroup J1 was found to be 2.4 fold under-represented in the 'HP' patients (p = 0.0035). These results are consistent with a previous observation made in Finnish T2DM patients. Moreover, assessing the haplogroup distribution in 'DP' versus 'HP' patients having diabetic siblings revealed that haplogroup J1 was virtually absent in the 'HP' group.

**Conclusion:**

These results imply the involvement of inherited factors, which modulate the susceptibility of haplogroup J1 to T2DM.

## Background

Mitochondrial dysfunction in Type 2 diabetes (T2DM) patients has been repeatedly reported [1], however clear evidence for mitochondrial genetic involvement in the disease is yet to be determined [2].

Recent studies revealed the apparent association of certain mitochondrial DNA (mtDNA) genetic backgrounds (haplotypes, haplogroups) with T2DM in Asians [3], but not in Caucasians [4,5]. Others described the association of certain mtDNA common variants, such as the T16189C variant with T2DM in various populations [6], however, this association was recently questioned [7]. Such apparent discrepancies are frequently viewed as pointing to false positive associations [8]. Nevertheless, putative functionality of mtDNA genetic variants could be strongly affected by population dynamics due to the exceptionally high genetic variability of human mtDNA, with many of the mtDNA SNPs being population specific [9,10]. This is evident when considering the association of mtDNA haplogroup J with longevity in the Irish, some French, and in northern *but not *southern Italians [11]. Other factors that add to the complex nature of mitochondrial genetics involvement in multifactorial diseases include: (A) disease-associated somatic mtDNA mutations; (B) the close interaction of mtDNA-encoded proteins with nuclear DNA-encoded proteins [12]; and (C) the possibility that many complex diseases, such as T2DM, constitute a mixture of disease entities. Accordingly, case-control association studies of mtDNA with T2DM as a whole might mask intermediary disease predisposing states, yielding average results with similar haplogroups distribution to that of a control population [10]. This interpretation is reflected in the lack of mtDNA haplogroups' association with T2DM that was reported in a study of more than 6,000 healthy and T2DM Caucasian samples [5]. Moreover, when we compared the mtDNA haplogroup distribution that was recently analyzed in a set of Ashkenazi Jewish T2DM patients [10] to 565 non diabetic Ashkenazi Jewish controls [13] we once again observed no significant differences in distribution of haplogroups between cases and controls (Additional file 1: Table S1, R × C test of independence performed on this data resulted in p = 0.1). To overcome this difficulty, and since the heritability of T2DM as a whole is not robust, we adopted a different approach, namely study the association of mtDNA haplogroups with T2DM in a cohort divided into sub-groups, according to the parental diabetes status, i.e. patients with healthy parents or T2DM parents.

## Methods

### Patient Cohorts and mtDNA haplogroup assignment

The assembly of T2DM patients' cohorts analyzed in the current study and the process of designating the mtDNA genetic haplogroups were previously described [10]. Briefly, the patients are all of Jewish origin, have no reported family connections, and were recruited in 7 different medical centers in Israel, following informed consent and approval by local IRB and national Helsinki medical research ethics boards. The studied cohort encompasses three Jewish subgroups: Ashkenazi (Ash), Sephardic (Seph) and North African Jews (NAF). The countries of origin are as follows; Ash cohort – Germany, Austria, Switzerland, former Czechoslovakia, Hungary, Yugoslavia, United Kingdom, Ireland, Poland, USA, Canada, Holland, Argentina, Ukraine, Russia, Latvia, Lithuania and Romania; Seph cohort – Cyprus, Bulgaria, Greece, Spain, Italy and Turkey; NAF cohort – Morocco, Algeria, Tunisia, Libya and Egypt. If the patient's country of origin was 'Israel', the patients' declared ethnicity was assigned (Ash, Seph or NAF). MtDNA haplogroup analysis was performed by RFLP of selected mtDNA polymorphic sites and partial sequence of the hypervariable region 1 of the mtDNA as described previously [10]. Out of the 1179 patients comprising our formerly studied cohort [10], we currently focused on 1055 patients having sufficient family history information. This information originated from questionnaires completed by the patients themselves, providing direct answers to questions about health parameters and specifically the T2DM status of each of the parents, siblings and descendants.

### Permutation test

In order to detect candidate mtDNA haplogroups in which the parental diabetic status of the patients is altered, we used permutation tests. These tests were performed using MATLAB (v.6.5) script: The parental diabetic status (a binary indicator variable, 0 – healthy parents and 1-parents with T2DM) was randomly assigned to patients with known haplogroups. Next, the proportion of patients in each haplogroup who have a diabetic parent was calculated, and the absolute difference (two-tailed test) between each of these values and the overall proportion of patients having a diabetic parent (i.e., the proportion of patients with at least one diabetic parent irrespective of their genetic background) was recorded. This procedure was repeated 10,000 times. P-values were estimated as the proportion of cases in which the absolute difference obtained during the permutations was equal to or greater than that found in the original dataset. The same procedure was performed to detect candidate haplogroups among patients further divided into (1) patients having at least one T2DM sibling and (2) patients with healthy siblings.

### Log linear model analysis and model selection procedure

Since our dataset include only categorical/strata variables we adopted a statistical method that allows analyzing contingency tables or frequencies of occurrence. Specifically, we used a Log-Linear Model analysis to assess the relationship between mtDNA haplogroups of the patients and the T2DM status of their parents (healthy vs. T2DM parents), with the T2DM parents further divided into T2DM mother only, T2DM father only and T2DM mother and father. We also took into account the T2DM status of the siblings, as well as geographic origin of each Jewish population.

As accepted in Log Linear analysis, higher-order interaction terms were examined first. The derived lower-order interaction terms were excluded since they are already included in the former. All examined models included the effects of the four main components outlined above. According to this logic, we first examined the four-way-interaction term model, followed by the models harboring different combinations of three- and two-way-interaction terms. The model best fitting our data was chosen using a model selection procedure [14]. Briefly, the model with the lowest Akaike Information Criterion (AIC) was considered as a reference model best explaining the data with a minimum of free parameters, and the rest were evaluated based on their AIC deviation (ΔAIC) from that model. Because AIC is a comparative index, only relative values are relevant. For log linear models, a relative AIC value, scaled so that AIC for the saturated model is zero, is given by AIC = *G*^2 ^- 2*(*DF*) where *G*^2 ^is the best-fit likelihood ratio statistic, testing the model against the saturated model, and *DF *is its degrees of freedom [15]. Both values (G^2^, DF) are reported by standard statistical software packages. As accepted in such an analysis, only the reference model as well as models with ΔAIC ≤ 2 were considered for further investigation [14]. Similar results were obtained both when we started with a saturated model harboring all possible interaction terms or when testing the contribution of each interaction to the overall significance by hierarchically removing different combinations of the tested components [16]. In this case, changes in *G *(ΔG values) were used to evaluate the significance of the different parameters or combinations that were removed from the model.

## Results

In order to test whether parental T2DM status influences the association of mtDNA haplogroups with T2DM, we compared the distribution of mtDNA haplogroups in T2DM patients that have either healthy or T2DM parents. This information was provided by the patients in response to a questionnaire (Methods section).

To this end, we first determined the mtDNA genetic backgrounds of 1055 T2DM patients of Jewish origin (661 Ashkenazi Jews [Ash] and 394 non-Ashkenazi Jews, comprising Sephardic [Seph] and North African Jews [NAF]), revealing that ~90% of the subjects belonged to one of the 12 most prevalent mtDNA haplogroups in Ashkenazi Jews, i.e., K1, K2, U (non-K), H, V, J1, J2, T, N1b, I, X, W. This cohort is a subset of a cohort of patients that we previously studied [10] (see Materials and Methods section).

Since the family history (including T2DM history of parents and siblings) and information regarding the geographic/ethnic origin was available for the subjects, patients were divided into the following: (A) a group with **at least one **diabetic parent (designated 'DP', further divided into three subgroups -only diabetic mother, only diabetic father and both diabetic parents) and (B) a group of patients of whom **neither **of the parents had T2DM (designated 'HP' e.g., healthy parents).

To avoid multiple testing we first identified candidate haplogroups for association with T2DM in the context of the parental health status using a permutation test. This analysis yielded a significant 2.4 fold under-representation of haplogroup J1 in the 'HP' versus 'DP' groups of patients (13/369, 3.5% in the 'HP' versus 57/686, 8.3% in the 'DP' group, p = 0.0035, Table 1). Such a significant difference was not observed in any other tested haplogroups. The observed 2.4 fold under-representation of haplogroup J1 in the 'HP' versus 'DP' groups of patients was also evident after dividing the total Jewish sample into Ashkenazi (Additional file 1: Table S2) and the smaller sub-group of non-Ashkenazi patients (Additional file 1: Table S3). Hence haplogroup J1 was selected as a candidate haplogroup for further investigation.

**Table 1:** Permutation test of haplogroup distribution among patients with T2DM parents (designated 'DP') versus patients with healthy parents (designated 'HP').

**Haplogroup**	**'HP' Patients**	**'DP' Patients**	**P value (permutation test)**	**Total patients**	**'DP'/Total**
H	87 (23.6%)	172 (25.1%)	0.604	259	0.664093
HV	38 (10.3%)	75 (10.9%)	0.7496	113	0.663717
J1	13 (3.5%)	57 (8.3%)	**0.0035**	70	0.814286
J2	7 (1.9%)	4 (0.6%)	0.0556	11	0.363636
K1	84 (22.8%)	122 (17.8%)	0.0608	206	0.592233
K2	17 (4.6%)	25 (3.6%)	0.5141	42	0.595238
N1b	21 (5.7%)	22 (3.2%)	0.0699	43	0.511628
Other	40 (10.8%)	76 (11.1%)	0.9203	116	0.655172
T	17 (4.6%)	40 (5.8%)	0.4726	57	0.701754
U	26 (7%)	51 (7.4%)	0.9025	77	0.662338
WXI	19 (5.1%)	42 (6.1%)	0.5807	61	0.688525
Total	369	686		1055	

Numbers in parentheses: percentage of the total sample size in each column.

To further decipher whether haplogroup J1 altered the susceptibility to T2DM depending on an additional factor and whether this added component is indeed inherited, a Log Linear Model analysis [16] was conducted (Table 2). We chose this approach as it offers a **single test **to assess the interaction of several factors that could affect the association of haplogroup J1 with T2DM, thus avoiding multiple consecutive tests. Specifically, this analysis tested interactions between the patients' genetic background and their T2DM family history, including the T2DM parental status, the existence of T2DM siblings and the geographic origin of the patients. These parameters were designated as J1 (patients assigned to haplogroup J1 versus patients pertaining to all other haplogroups in the aggregate); I ('HP' vs. 'DP' patients, with the latter further divided into three subgroups -only diabetic mother, only diabetic father and both diabetic parents); S (patients with at least one T2DM sibling vs. patients with no T2DM siblings) and P (population-of-origin including ASH, SEPH and NAF, noting differences in mtDNA genetic landscape among different Jewish populations, [10,17-20]).

**Table 2:** A log linear model analysis testing for the relationship between mtDNA genetic background (haplogroup J1 versus all other haplogroups in the aggregate), T2DM status of the parents (I), T2DM status of the siblings (S) and population-of-origin (P).

**Model No.**	**Model**	**G**^2^	**Degrees of Freedom**	Akaike Information Criterion (AIC)*	**ΔAIC**
1	J1 × I × S × P	72.7317	34	4.7317	43.0524
2	J1 × I × S; J1 × I × P; J1 × S × P; I × S × P	35.8467	23	-10.1533	28.1674
3	J1 × I × S; J1 × I × P; J1 × S × P	44.1794	29	-13.8206	24.5001
4	J1 × I × S; J1 × S × P	60.9351	35	-9.0649	29.2558
5	J1 × S × P	86.7039	38	10.7039	49.0246
6	J1 × S × P; J1 × I; S × I	44.1896	32	-19.8104	18.5103
7	J1 × I × S; S × P	47.2893	35	-22.7107	15.61
8	J1 × I × P; I × S; S × P	26.3029	29	-31.6971	6.6236
9	I × S × P; J1 × I	88.7164	31	26.7164	65.0371
10	J1 × I; J1 × S; J1 × P; I × S; I × P; S × P	21.1262	23	-24.8738	13.4469
11	J1 × I; J1 × P; I × S; S × P	21.6793	30	-38.3207	0
12	J1 × I; S × P; I × S	29.6666	32	-34.3334	3.9873

Using a model selection procedure [14] we found that the model best fitting our data included the following four two-way-interaction terms: J1 × I; J1 × P; I × S; S × P (Table 2, model #11). This model indicates that even after taking into account the significant under-representation of haplogroup J1 in the NAF population (J1 × P interaction term; ΔG = 7.99, DF = 2, p = 0.018) (Additional file 1: Table S4), and the significant variation in the number of patients with at least one T2DM sibling among the different populations (S × P interaction term; ΔG = 48.98, DF = 2, p < 0.001), there remains a significant interaction between haplogroup J1 and the parental T2DM status (J1 × I interaction term; ΔG = 12.23, DF = 3, p = 0.007). As expected, the analysis also indicated a significant interaction between the parental T2DM status and that of the T2DM siblings (I × S interaction term; ΔG = 30.44, DF = 3, p < 0.001).

The above findings further indicate that the association of haplogroup J1 with T2DM markedly depends on the T2DM status of the parents of the diabetic subjects. To substantiate this information we assessed the association of haplogroup J1 with the T2DM status of the patients' siblings. This was performed based on the assumption that mtDNA is maternally inherited and therefore all biological siblings of a given patient will carry the same mtDNA genetic background. Therefore, the T2DM history of the patient's siblings could substantiate the dependency of haplogroup J1 association with the parental disease-status. Since T2DM is an age-related disease and because the average age of our T2DM patients was 55 years old, only siblings of age above 54 years were included in the analysis. Moreover, the distribution of the number of siblings above 54 years of age was consistent among haplogroups (J1 vs. the aggregate of all other haplogroups) and between the 'DP' and 'HP' patient groups (G = 2.799, DF = 5, p = 0.731; and G = 8.038, DF = 5, p = 0.154, respectively). Thus, we could divide our patient group into patients with at least one T2DM sibling and patients with healthy siblings. This division significantly emphasized the under-representation of haplogroup J1 in the 'HP' patients as was clearly evident in our statistical analysis (J1 × I interaction term; ΔG = 12.23, DF = 3, p = 0.007; Table 2). Furthermore, haplogroup J1 was virtually absent among 'HP' patients with T2DM siblings (1/88, 1.1%), but retained its proportion in the 'DP' patients with at least one T2DM sibling (23/279, 8.2%, p = 0.022) (Table 3). Notably, the percentages of patients with at least one T2DM sibling remained unaltered among the 'HP' and 'DP' T2DM patients pertaining to all other haplogroups. When the same analysis was performed in patients with healthy siblings, J1 was under-represented to the same extent as evident in the entire cohort. This suggests that the T2DM status of the parents carries a modifying effect of haplogroup J1 association with T2DM. It is important to note that although T2DM siblings appear to enhance the association of J1 with T2DM family history, the "sibling" component failed to produce a significant interaction with J1 on its own (Table 2). We interpret this as the "sibling" factor in our analysis being a dependent component of the T2DM parental status, as intuitively could be expected from the fact that the same mtDNA is transferred to all of the siblings; thus, its effect could be revealed only when combined with the parental T2DM status, further supporting the interaction of T2DM family history with haplogroup J1. Taken together, our approach proved successful in demonstrating a significant mitochondrial genetic contribution to T2DM, which heretofore has been difficult to tease out from among the multiple factors contributing to T2DM disease predisposition.

**Table 3:** Permutation test of haplogroup distribution in 'HP' and 'DP' patients with at least one T2DM sibling.

**Haplogroup**	**'HP' patients with T2DM sibs**	**'DP' patients with T2DM sibs**	**P value (permutation test)**	**Total number of patients**	**'DP'/Total**
H	19 (21.6%)	73 (26.2%)	0.3997	92	0.793478
HV	13 (14.8%)	34 (12.2%)	0.5837	47	0.723404
J1	1 (1.1%)	23 (8.2%)	**0.0228**	24	0.958333
J2	2 (2.3%)	2 (0.7%)	0.246	4	0.5
K1	19 (21.5%)	39 (13.97%)	0.095	58	0.672414
K2	4 (4.5%)	15 (5.4%)	0.7962	19	0.789474
N1b	4 (4.5%)	9 (3.2%)	0.7448	13	0.692308
Other	8 (9.1%)	32 (11.5%)	0.5695	40	0.8
T	3 (3.4%)	14 (5%)	0.5842	17	0.823529
U	7 (7.9%)	21 (7.5%)	1	28	0.75
WXI	8 (9.1%)	17 (6.1%)	0.3475	25	0.68
Total	88	279		367	

Numbers in parenthesis: percentage of the total sample size in each column.

## Discussion

MtDNA association with T2DM has been described in Asians but was questioned in Caucasians. We hypothesized, that apart from the evident environmental differences, such inconsistency could reflect either of two alternatives: (A) mtDNA has little or no genetic contribution to T2DM in Caucasians or (B) mtDNA contribution to T2DM involves additional genetic factors, the contribution of which is more prominent in Caucasians, thus masking its association. We aimed to test the second possibility by investigating the distribution of mtDNA haplogroups in a large Jewish T2DM patient cohort taking into account the T2DM status of the patients' parents, thus controlling for the involvement of additional putative genetic factors. By using the Log Linear Model and model selection approach we were able to test for possible association of mtDNA haplogroups with T2DM taking into account not only the diabetes status of the parents as well as their siblings, but also controlling for the geographic origin of the studied populations in a single test. Our approach revealed an association of haplogroup J1 with T2DM depending on the diabetes status of the parents. Most importantly, the fact that the three way interaction term J1 × I × P was not included in the emerging model indicated that the under-representation of haplogroup J1 in the 'HP' versus 'DP' groups of patients was consistent among the studied Jewish populations. This result implies that the interaction J1 × I might be applicable to populations other than Jews. Indeed, Mohlke et al previously reported an association of haplogroup J with T2DM in Finns while controlling for the parental diabetes status [4]. Thus, our careful control for family history information unearthed the association of mtDNA variants with T2DM, as was suggested for a non-Jewish population, supporting possible application of our approach to other populations of Caucasian ancestry.

A maternal bias towards the inheritance of T2DM has been debated [21,22]. Therefore, in our Log Linear Model, we divided the group of 'DP' patients into three subgroups – patients with T2DM mother only, patients with T2DM father only and patients with both T2DM parents. Within the 'DP' patients as a whole, the proportion of T2DM mothers exceeded by 3 fold that of T2DM fathers. However, this pattern was consistent in all patients, either pertaining to haplogroup J1 or non-J1 (38/57, 66.7% and 365/628, 58.1%, respectively), implying that there was little or no differential contribution of the parental gender to the altered distribution of J1 haplogroup in our patient cohort. In other words, the frequency of haplogroup J1 varied between 'HP' and 'DP' patients, rather than among the three 'DP' subgroups, resulting in a significant J1 × I interaction term. This implies that the modulating factor of haplogroup J1 association with T2DM is not necessarily maternally inherited.

Haplogroup J was previously associated with reduced tendency of certain Caucasian populations to develop Parkinson's disease [23] and with successful longevity [24]. Its sub-group J1 increases the penetrance of Leber's Hereditary Optic Neuropathy mutations, and recently was found by us to increase the susceptibility of Ashkenazi T2DM patients to develop certain complications [10]. This suggests that mutations defining this haplogroup likely have mitochondrial functional consequences. Our current association of haplogroup J1 with T2DM in Jews further underlines the phenotypic impact of this mtDNA genetic background.

Taken together our results suggest that haplogroup J1 alters the susceptibility to T2DM, depending on the parental health condition. This implies the existence of an inherited factor, which modulates haplogroup J1 association with T2DM. This very effect was more prominent when taking into account the siblings' T2DM status. A recent study that focused on certain mtDNA variants using Indian populations implied that certain combinations of nuclear and mitochondrial encoded variants, especially in regulatory factors of mitochondrial biogenesis, can increase the susceptibility to T2DM, thus supporting our interpretation [12]. Moreover, the synergistic effect of nuclear-mitochondrial genetic variants on disease susceptibility was shown in other disorders as well [25]. Thus, mitochondrial genetic association with complex diseases almost certainly involves the interaction of nuclear and mtDNA-encoded factors.

## Conclusion

Several lines of evidence have emerged from our findings, all stem from the use of a suitable approach to identify association of mtDNA haplogroups with T2DM in Caucasian (Jewish) patients:

1. Our approach employs a comparison between two subgroups within a large heterogeneous Jewish cohort of T2DM patients classified according to the diabetes status of their parents. Similar to our previous report [10], the current analysis suggests that, rather than comparing the entire cohort of diabetic patients to healthy individuals, a comparison of subgroups within the diabetic population is more meaningful. This is because it enables focusing on distinct phenotypes within the heterogenic spectrum of the diabetic syndrome, thus providing an approach to sort mtDNA variants' association with T2DM from the 'noise'.

2. Our comparisons yielded a significant association of mtDNA haplogroup J1 with T2DM depending on the parental diabetes status. More specifically, haplogroup J1 was notably under-represented in T2DM patients with healthy parents, as compared to patients with T2DM parents. The interpreted under presentation of haplogroup J1 in the HP patients is in light of the fact that the prevalence of haplogroup J1 in the general Ashkenazi Jewish population was similar to the DP patients. Apparently, it seems that the penetrance of the phenotypic effect of mtDNA variants is differentially modified by parentally inherited factors. Since such modifying factors were not taken into account in previous mtDNA association studies with T2DM, they could provide a possible explanation why past comparisons of mtDNA variation between Caucasian T2DM patients and controls failed to yield clear association.

3. Our approach succeeded in detecting the existence of an inherited component, which affected the association of mtDNA haplogroup J1 with T2DM in Caucasians. Detecting the effect of such a component became feasible by utilizing the Log Linear model and model selection approach, which controlled for the geographic origin of the patients, thus ruling out the involvement of population sub-structure in the observed association. In addition, this approach allowed in a single test to assess whether any of the multiple parameters (geographic origin of the patients, the existence of T2DM siblings and parental sex) affect the association while avoiding multiple testing.

4. Our model selection method showed that the observed association depended on the diabetes status of the parents regardless of their sex. This implies the existence of modifying factor/s for the phenotypic effect of haplogroup J1 that is inherited either from the father or the mother, and is therefore most plausibly located in the nuclear genome.

In summary our discovered association of mtDNA variants with T2DM in Caucasians support our approach and suggest that during mtDNA association studies the involvement of other genetic factors should be controlled for.

## List of abbreviations

T2DM: (Type 2 Diabetes mellitus); mtDNA: (mitochondrial DNA); HP: patients with both healthy parents; DP: patients with at least one diabetic parent.

## Competing interests

The authors declare that they have no competing interests.

## Authors' contributions

JF carried out the molecular genetic studies, statistically analyzed the data and participated in drafting the manuscript. OO designed the statistical approach. IB participated in the data collection. JC collected patients' samples. JW collected patients' samples. IHB collected patients' samples. BG collected patients' samples, participated in study design and in drafting the manuscript. DM designed the study, participated in data analysis and wrote the manuscript. All authors approved the final manuscript.

## Pre-publication history

The pre-publication history for this paper can be accessed here:



## Supplementary Material

Additional file 1 **Tables S1-S4**. Table S1: Haplogroup Distribution of random controls versus T2DM patients. Table S2: Permutation test of haplogroup distribution in 'HP' and 'DP' patients of the Ash population. Table S3: Permutation test of haplogroup distribution in 'HP' and 'DP' patients of the Non-Ashkenazi patients (Seph +NAF). Table S4: Distribution of patients pertaining of haplogroup J1 versus those pertaining to other haplogroups in the three patients populations. Click here for file
