# Supplementary material for: Parental diabetes status reveals association of mitochondrial DNA haplogroup J1 with type 2 diabetes
Source: BMC Med Genet. 2009 Jun 18;10:60. doi: 10.1186/1471-2350-10-60 (PMC2706816; doi:10.1186/1471-2350-10-60)
Supplement: Additional file 1 — Tables S1-S4. Table S1: Haplogroup Distribution of random controls versus T2DM patients. Table S2: Permutation test of haplogroup distribution in 'HP' and 'DP' patients of the Ash population. Table S3: Permutation test of haplogroup distribution in 'HP' and 'DP' patients of the Non-Ashkenazi patients (Seph +NAF). Table S4: Distribution of patients pertaining of haplogroup J1 versus those pertaining to other haplogroups in the three patients populations. [file 1471-2350-10-60-S1.doc]

**Additional file 1**

Table S1: Haplogroup Distribution of random controls versus T2DM patients (Ashkenazi Jewish dataset).

| **Haplogroup** | **Control *** | **T2DM (ASH)** | **Total** |
| --- | --- | --- | --- |
| **U (nonK)** | 32 | 51 | **83** |
| **K** | 181 | 222 | **403** |
| **HV** | 50 | 74 | **124** |
| **H** | 115 | 175 | **290** |
| **J1** | 37 | 57 | **94** |
| **J2** | 3 | 2 | **5** |
| **T** | 27 | 28 | **55** |
| **N1b** | 57 | 44 | **101** |
| **WXI** | 19 | 31 | **50** |
| **Other** | 44 | 76 | **120** |
|  |  |  |  |
| **Total (N)** | **565** | **760** | **1325** |

*The control dataset stem from: Behar DM, Hammer MF, Garrigan D, Villems R, Bonne-Tamir B et al. (2004) MtDNA evidence for a genetic bottleneck in the early history of the Ashkenazi Jewish population. Eur J Hum Genet 12(5): 355-364.

Table S2: Permutation test of haplogroup distribution in 'HP' and 'DP' patients of the Ash population.

| **Haplogroup** | **'HP' Patients** | **'DP' Patients** | P value  (permutation test) | Total patients | 'DP'/  Total |
| --- | --- | --- | --- | --- | --- |
| H | 48 | 97 | 0.4409 | 145 | 0.668966 |
| HV | 22 | 43 | 0.7849 | 65 | 0.661538 |
| J1 | 10 | 39 | **0.0199** | 49 | 0.795918 |
| K1 | 69 | 89 | **0.0227** | 158 | 0.563291 |
| K2 | 16 | 23 | 0.6059 | 39 | 0.589744 |
| N1b | 17 | 20 | 0.2187 | 37 | 0.540541 |
| Other | 27 | 40 | 0.5001 | 67 | 0.597015 |
| T | 5 | 21 | 0.094 | 26 | 0.807692 |
| U | 14 | 32 | 0.4369 | 46 | 0.695652 |
| WXI | 9 | 18 | 0.8457 | 27 | 0.666667 |
| **Total** | **237** | **422** |  | **659** |  |

*missing 2 J2 patients – were not included in the initial analysis.

Table S3: Permutation test of haplogroup distribution in 'HP' and 'DP' patients of the Non-Ashkenazi patients (Seph +NAF).

| Haplogroup | **'HP' Patients** | **'DP' Patients** | P value  (permutation test) | Total patients | 'DP'/Total |
| --- | --- | --- | --- | --- | --- |
| H | 39 | 75 | 0.8084 | 114 | 0.657895 |
| HV | 16 | 32 | 1 | 48 | 0.666667 |
| J1 | 3 | 18 | 0.0938 | 21 | 0.857143 |
| J2 | 5 | 4 | 0.161 | 9 | 0.444444 |
| K | 16 | 35 | 0.8767 | 51 | 0.686275 |
| N1b | 4 | 2 | 0.0897 | 6 | 0.333333 |
| Other | 13 | 36 | 0.3231 | 49 | 0.734694 |
| T | 12 | 19 | 0.5457 | 31 | 0.612903 |
| U | 12 | 19 | 0.5504 | 31 | 0.612903 |
| WXI | 10 | 24 | 0.7061 | 34 | 0.705882 |
| **Total** | **130** | **264** |  | **394** |  |

Table S4: Distribution of patients pertaining of haplogroup J1 versus those pertaining to other haplogroups in the three patients populations. Percentage of J1 patients in each population are shown in parenthesis.

| **Population** | **J1** | **Non-J1** | **Total** |
| --- | --- | --- | --- |
| **Ash** | 49 (7.4%) | 611 | 660 |
| **Seph** | 15 (8.2%) | 167 | 182 |
| **NAF** | 6 (2.8%) | 205 | 211 |
| **Total** | 70 | 983 | 1053 |
